# β-aminobutyric acid (BABA)-induced resistance to tobacco black shank in tobacco (*Nicotiana tabacum L.*)

**DOI:** 10.1371/journal.pone.0267960

**Published:** 2022-06-09

**Authors:** Xiyue Ren, Jianjun Wang, Faliang Zhu, Zhijiang Wang, Jian Mei, Yonghui Xie, Tao Liu, Xianwen Ye

**Affiliations:** 1 College of Agronomy and Biotechnology, Yunnan Agricultural University, Kunming, China; 2 National-Local Joint Engineering Research Center on Germplasms Innovation & Utilization of Chinese Medicinal Materials in Southwest China, Yunnan Agricultural University, Kunming, China; 3 Yunnan Tobacco Co., Ltd., Kunming Branch, Kunming, China; University of Agriculture Faisalabad, PAKISTAN

## Abstract

Tobacco black shank is a kind of soil-borne disease caused by the Oomycete Phytophthora parasitica. This disease is one of the most destructive diseases to tobacco (Nicotiana tabacum L.) growth worldwide. At present, various measures have been taken to control this disease, but they still have different challenges and limitations. Studies have shown that β-aminobutyric acid (BABA), a nonprotein amino acid, can enhance disease resistance in plants against different varieties of pathogens. However, it is unclear whether BABA can induce plants to resist *Phytophthora parasitica* infection. Therefore, this study aims to explore the effect and related mechanism of BABA against tobacco black shank. Our results showed that 5 mmol^.^L^-1^ BABA had an obvious anti-inducing effect on the pathogenic fungus and could effectively inhibit the formation of dark spots in the stems. The results also showed that a large amount of callose deposition was observed in BABA-treated tobacco. Furthermore, the application of BABA induced the accumulation of H_2_O_2_ in tobacco and effectively regulated the homeostasis of reactive oxygen in tobacco plants, reducing the toxicity of H_2_O_2_ to plants while activating the defense system. In addition, BABA spray treatment could induce an increase in the concentrations of salicylic acid (SA) and jasmonic acid-isoleucine (JA-Ile) in tobacco, and the gene expression results confirmed that BABA upregulated the expression of SA-related genes (*PR1*, *PR2* and *PR5*), JA-related genes (*PDF1*.*2*) and ET-related genes (*EFE26* and *ACC oxidase*) in tobacco plants. Taken together, BABA could activate tobacco resistance to black shank disease by increasing H_2_O_2_ accumulation, callose deposition, plant hormone (SA and JA-Ile) production, and SA-, JA-, and ET- signaling pathways.

## 1. Introduction

Plants increase their resistance to diseases after being stimulated by biotic and abiotic factors. When plants are later attacked by the same disease, they can have a stronger and faster defense response, which is called plant-induced disease resistance. Generally, plant-induced disease resistance is divided into systemic acquired resistance (SAR) and induced systemic resistance (ISR) [1]. Of the various categories of inducers, chemical inducers are environmentally safe and well documented for inducing long-lasting resistance. Moreover, chemical inducers do not produce chemical-tolerant pathogen strains or the breakdown of plant resistance [2]. For example, methyl jasmonate (Me-JA) [3], salicylic acid (SA) [4], benzo-(1,2,3)-thiadi S-methyl oxazole-7-carbosulfate (BTH) [5] and other chemical inducers can induce resistance against a variety of pathogens. β-Aminobutyric acid (BABA), a nonprotein amino acid, can induce systemic acquired resistance in multiple plants [6], such as tobacco [7], Arabidopsis [8], tomato [9] and cauliflower [10]. In recent years, it has been found that 10 mmol^.^L^-1^ BABA can effectively induce grape resistance to *Botrytis cinerea* [11]. In addition, BABA application improves soybean resistance to aphids through the activation of phenylpropanoid metabolism and callose deposition [12]. Therefore, as an efficient and broad-spectrum inducer, BABA has great development potential in the treatment of plant diseases.

Tobacco plays an important role in the Chinese national economy. However, fungal diseases have greatly influenced the quality and yield of tobacco. Tobacco black shank is a kind of soil-borne disease caused by *P*.*parasitica* [13]. Pathogens mainly damage the stem base and roots of adult plants, causing large economic losses to tobacco production every year [14]. In recent years, tobacco black shank has become increasingly serious because of the influence of climate change, continuous cropping and fertilization conditions. In production, the disease is generally controlled by planting disease-resistant varieties and chemical pesticide control measures. Although these methods have a certain control effect, they also have some disadvantages, such as weakened resistance of disease-resistant varieties, pesticide residues, and drug resistance of pathogenic bacteria. With the development of nonpolluting tobacco production, the use of inducers is an ideal way to control tobacco diseases and develop plant resistance mechanisms. To date, there is no research report about the effect of BABA on tobacco black shank. Therefore, we used tobacco and *Phytophthora parasitica* to explore the effect and intrinsic mechanism of BABA application in tobacco resistance against tobacco black shank. The purpose of this study was to explore the ideal drugs and control methods for tobacco black shank and to provide a scientific basis for their practical application in the field.

## 2. Materials and methods

### 2.1 Experimental site and experimental materials

The experimental tobacco cultivar Yunyan 87 was used here, and the experimental strain was *P*. *parasitica*. Tobacco plants were grown in the greenhouse of Yunnan Agricultural University in northern Kunming (25°7’N, 105°45’E, 1 960 m).

### 2.2 Determination of the direct bacteriostatic effect of BABA on *P*. *parasitica*

Make Reference to that method for determining the toxicity of bactericide-growth rate method [15] *P*. *parasitica* was cultured on potato dextrose agar (PDA) medium. A 6 mm diameter bacterial disk was cut with a hole punch and then placed using tweezers onto PDA plates containing 0, 1, 5, 10, 20 mmol^.^L^-1^ BABA for inoculation, with 0 mmol^.^L^-1^ BABA PDA plates used as a control. Three replicates were used per concentration. The cells were cultured at 30°C for 2–7 days, and the growth of mycelia was observed during this period. The cross method was used to measure the colony diameter and calculate the inhibition rate. Inhibition rate = (controlled colony diameter-treated colony diameter)/controlled colony diameter×100% (1)

### 2.3 Experimental design

#### 2.3.1 Effects of BABA treatment on leaf phenotype

This study used a floating seedling system to raise tobacco seeds. When the tobacco plants grew to the seedling stage, they were transferred to a plastic bowl filled with sterilized nutrient soil. When the tobacco plants grew to the 5–6 leaf stage, 1, 5, 10, and 20 mmol^.^L^-1^ BABA were sprayed on the leaf surface, and distilled water was used as the control. Three replicates were used per concentration. Leaf phenotypes were observed after three days.

#### 2.3.2 Effects of BABA on tobacco black shank in a greenhouse

Selected plants with consistent growth were then sprayed with distilled water and 5 mmol^.^L^-1^ BABA. Spraying was performed three times total at once every three days. *P*. *parasitica* was inoculated three days after the last spray. The incidence of tobacco black shank was observed and recorded. The following is a classification of the severity of tobacco black shank disease. Level 0: The whole plant is disease-free. Level 1: The diseased spot on the stem does not exceed one-third of the stem circumference, or less than one-third of the leaves are wilted. Level 3: The diseased spots on the stem surround one-third to one-half of the stem circumference, or one-third to one-half of the leaves are slightly withered, or a few spots appear on the lower part of the leaves. Level 5: The diseased spots on the stem exceed one-half of the stem circumference, but do not cover the entire stem circumference, or one-half to two-thirds of the leaves are wilted. Level 7: The diseased spots on the stem surround the stem circumference, or more than two-thirds of the leaves are wilted. and Level 9: The diseased plants are considered dead.

#### 2.3.3 Physiological, biochemical and molecular mechanisms of BABA-induced resistance to black shank disease in tobacco

When the tobacco plants grew to the 5–6 leaf stage, 5 mmol^.^L^-1^ BABA or distilled water was sprayed on the surface of the leaves. Half of the tobacco plants with different spray treatments were inoculated with mycelial pellets of *P*. *parasitica* at the base of the wounded stem. Four days after inoculation, tobacco leaves at the same stage were collected from plants undergoing various treatments and stored in the refrigerator at -80°C for testing after quickly freezing them in liquid nitrogen.

### 2.4 Detection of callose and H2O2 content

The callose deposition was measured based on a protocol reported by Lin Jin [16]. The tobacco leaves were stained for callose by aniline blue. The callose deposition was fluorescent microscope and further quantified by Image J software. Each treatment included three plants.

The H_2_O_2_ detection: A total of 0.1 g of sample was weighed, ground evenly with 1 mL of cold acetone, and centrifuged at 12 000 rpm for 10 min (4°C). A total of 1 mL of supernatant was collected to determine the absorbance at 415 nm according to the method of Patterson [17], with units of μmol^.^g^-1^. The results were measured based on fresh weight.

### 2.5 Determination of and plant hormones

The ethylene (ET) content was determined by gas chromatography with the following parameters: chromatographic column: capillary column; carrier gas speed: 1 mL^.^ min^-1^; injection volume: 5 μL; injection port temperature: 130°C; detector temperature: 230°C; and column temperature: 80°C. After the instrument was ready, the sample and standard were injected into the gas chromatograph for analysis. Based on the retention time of the peak of the standard solution, the ethylene content measured in the sample was calculated from the peak area.

Enzyme-linked immunosorbent assay (ELISA) was used to detect the levels of salicylic acid (SA), jasmonic acid (JA), jasmonic acid-isoleucine (JA-Ile) and abscisic acid (ABA). (1) Reagents, samples and standards were prepared; (2) prepared samples and standards were reacted at 37°C for 30 minutes; (3) the plate was washed 5 times, followed by the addition of enzyme-labeled reagent and reaction at 37°C for 30 minutes; (4) the plate was washed 5 times and colored liquids A and B were reacted at 37°C for 10 minutes; (5) termination liquid was added; (6) the OD450 value was read within 15 minutes; and (7) the linear regression equation of the standard curve was calculated with the concentration of the standard substance and the OD value. Substituting the OD value of the sample into the equation, the sample concentration was calculated by multiplying the dilution multiples to obtain the actual concentration of the sample.

### 2.6 Determination of NADPH oxidase gene and plant hormone-related genes

The conventional TRIzol method was used to extract and purify RNA. The first strand of cDNA was reverse transcribed and used as a template for RT–PCR. Real-time quantitative PCR was carried out in a real-time quantitative PCR detection system. The 20 μL reaction system contained 2 μL of cDNA, 10 μL of SYBR Green supermix solution, 0.8 μL of upstream and downstream primers, and 7.2 μL of ddH_2_O. The basic PCR procedure was as follows: predenaturation at 95°C for 10 min, followed by 40 cycles of denaturation at 95°C for 15 s, annealing at 60°C for 30 s, and extension at 72°C for 30 s. Fluorescence data were collected at the end of annealing stage within each cycle. The calculated internal standard was the fluorescence value of the ACTIN gene in tobacco, and the relative expression level of the gene was calculated according to the 2^-ΔΔC(T)^ method of Livak and Schmittgen [18] with three replicates. The specific primer sequences in Table 1 were used for amplification of genes.

**Table 1:** Genes and primers used in real-time PCR analysis.

Gene	Forward primer(5’-3’)	Reverse primer(5’-3’)
*Actin*	ACTGGTGTTATGGTTGGTATGGGTC	ATGACCTGCCCATCTGGTAACTC
*PR1*	TTCTCTTTTCACAAATGCCTTC	CACCTGAGTATAGTGTCCACAC
*PR2*	CACCATTTGTTGCTCCTG	ATTCGCTAAGATCCCTGA
*PR5*	GCTTCCCCTTTTATGCCTTC	CCTGGGTTCACGTTAATGCT
*PDF1*.*2*	GCGCCGGTATTTTTATATTATTGTAACAACAA	GCGCCACACAACACATACATCTATACATTG
*NtrbohD*	TGGAGGAAATAATTTCAATACAAGG	GCATCACAACCACAACTATATATCAAC
*ABI4*	TACTCTACGGCTCAAGGGCT	TTGTGTGGAAGACGAGGAGC
*ABA2*	GCTTCCTGCTGGGAATAGCA	CGCTCGGTATCTTCTGCCTT
*EFE26*	CGGACGCTGGTGGCATAAT	CAACAAGAGCTGGTGCTGGATA
*ACC Oxidase*	GACAAAGGGACATTACAAGAAGT	GAGAAGGATTATGCCACCAG

### 2.7 Statistical analyses

The data obtained from these experiments were statistically analyzed by Excel 2013 software. One-way analysis was used to analyze the difference significance, and GraphPad Prism software was used to draw the graph.

## 3. Results

### 3.1 BABA has a direct antibacterial effect on *P*. *parasitica*

**Fig 1:**
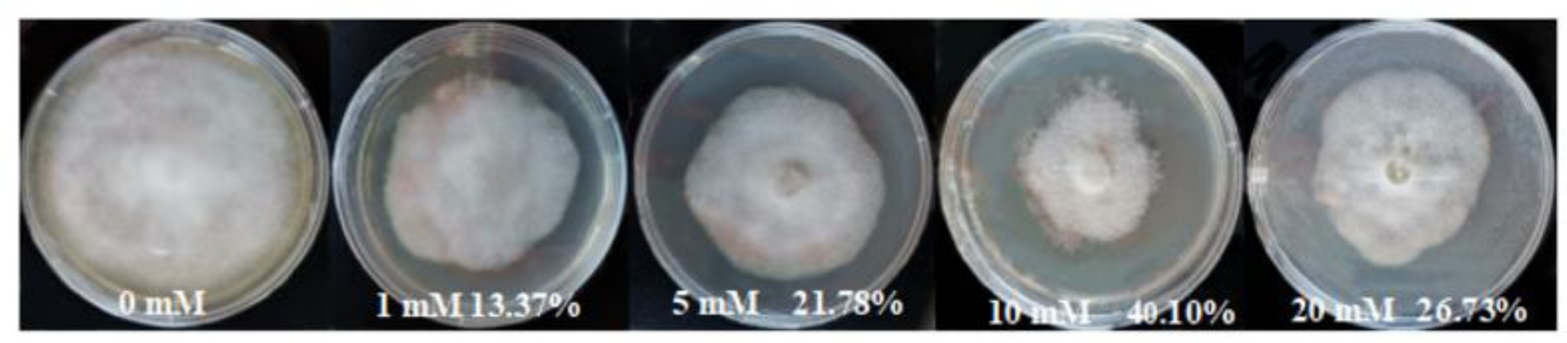
*P*. *parasitica* cultures in PDA medium illustrating that the inhibition of mycelial radial growth is affected by BABA at various concentrations. The mycelial colonies were six days old.

After *P*. *parasitica* was cultured for 6 days, it was found that BABA had an inhibitory effect on the growth of *P*. *parasitica* hyphae. The *P*. *parasitica* colony diameter decreased with increasing BABA concentration (Fig 1). *P*. *parasitica* was inhibited by 21.78% at 5 mmol^.^L^-1^ BABA; *P*. *parasitica* was inhibited by 40.10% at 10 mmol^.^L^-1^ BABA. However, *P*. *parasitica* was inhibited by only 26.73% at 20 mmol^.^L^-1^ BABA (Table 2). BABA had extremely significant effect on the inhibition rate of *P*. *parasitica*.

**Table 2:** Inhibition rate of BABA on *P*. *parasitica* growth.

C(BABA)/(mmol^.^L^-1^)	Colony diameter(cm)	Inhibition rate (%)	Remarkable analysis
0	6.73	0	
1	5.83	13.37	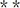
5	5.27	21.78	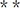
10	4.03	40.10	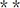
20	4.93	26.73	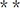

### 3.2 Phenotypic comparison of tobacco treated with different concentrations of BABA and *P*. *parasitica*

**Fig 2:**
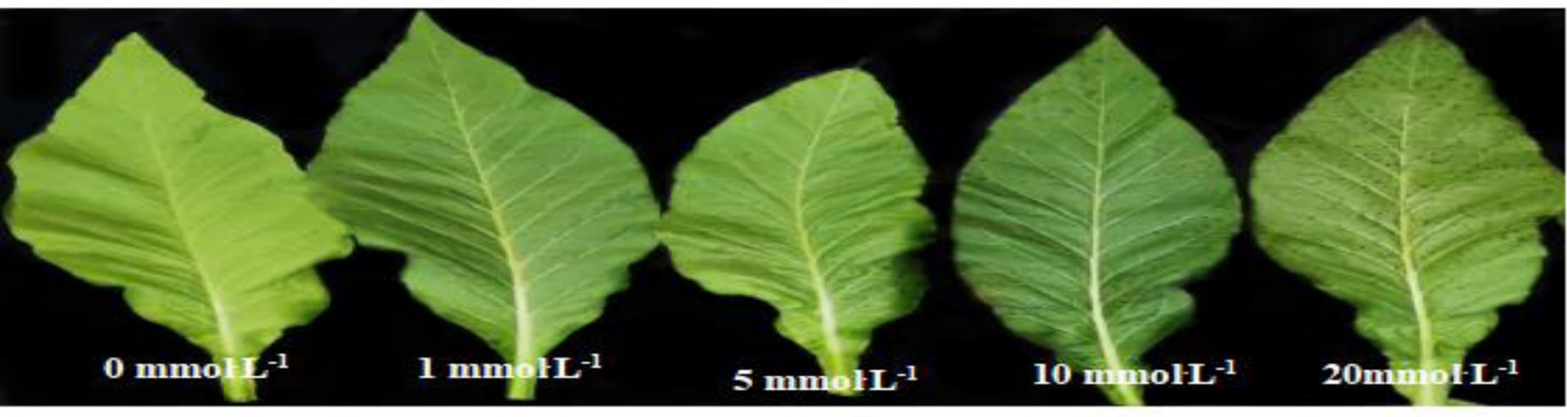
Phenotype of tobacco leaves sprayed with different concentrations of BABA.

Spraying BABA on plants caused necrotic spots on leaves, which represents a response of the plants to external stresses (irritation) and induces plants to enter a defensive state. Tobacco leaves were sprayed with 0 (water), 1, 5, 10 and 20 mmol^.^L^-1^ BABA solutions. After three days, there was no obvious change in tobacco leaves sprayed with water and 1 mmol^.^L^-1^1 BABA. When the concentration of BABA was 5 mmol^.^L^-1^, a small number of spots appeared on the tobacco leaves. A large number of spots appeared on the tobacco leaves when the concentrations of BABA were 10 mmol^.^L^-1^ and 20 mmol^.^L^-1^ (Fig 2). The results showed that the number and size of spots were positively correlated with the concentration of BABA. When the concentration of BABA is high, it will cause great damage to tobacco leaves.

**Fig 3:**
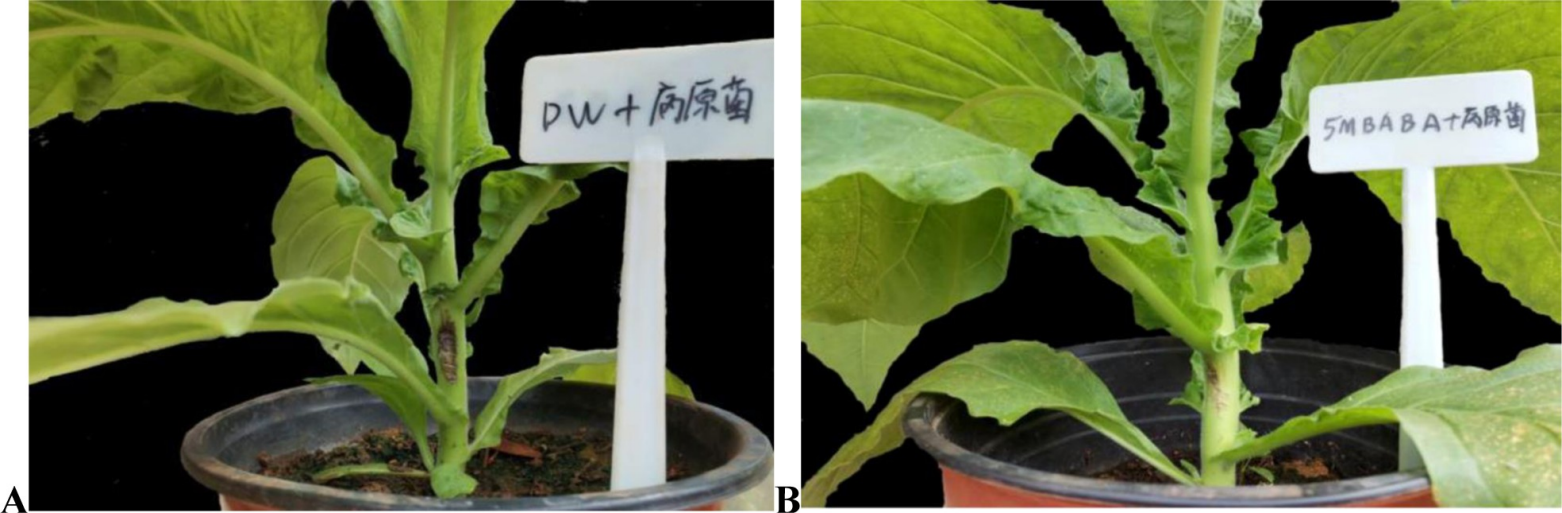
Three days after the tobacco plant was inoculated with *P*. *parasitica*, the symptoms of dark spots on the stem of the tobacco plant appeared. (A) Water + *P*. *parasitica* treatment. (B) 5 M BABA + *P*. *parasitica* treatment.

In this experiment, three days after tobacco inoculated with *P*. *parasitica*, Water + *P*. *parasitica* treatment had a disease severity of grade 3, and the color depth and area of black spots were obvious. 5 M BABA + *P*. *parasitica* treatment had a black shank disease severity of grade 1, and the dark spots are not obvious (Fig 3). This result shows that 5 mmol^.^L^-1^ BABA can effectively reduce the effect of *P*. *parasitica* on tobacco.

### 3.3 Callose in tobacco

**Fig 4:**
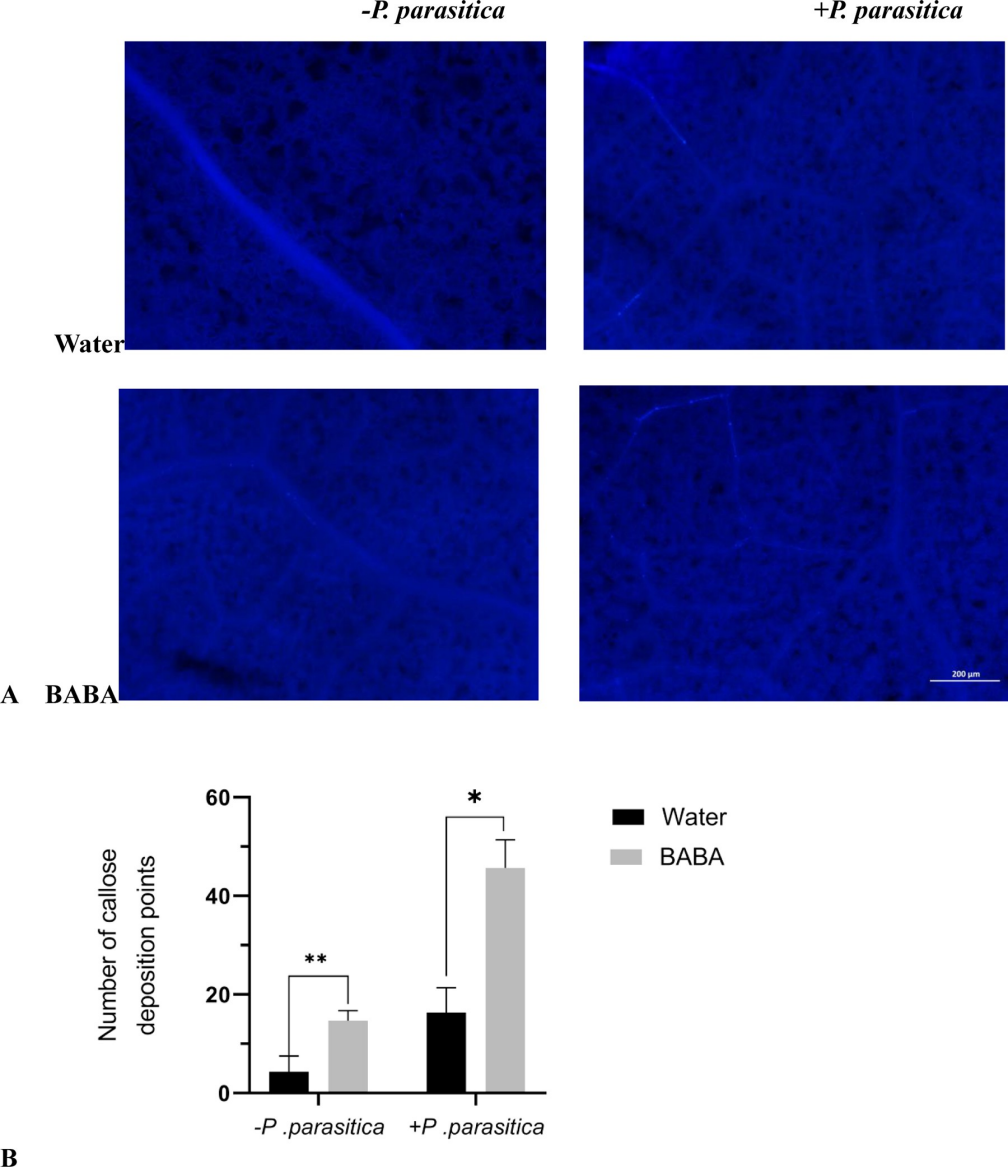
The effect of BABA on the callose deposition of tobacco. (A) The images of callose deposition in tobacco leaves were taken by fluorescence microscope. (B) The number of callose deposition points was counted by the Image J software. Note: The error line represents the standard deviation (*n* = 3), * represents a significant difference at *P* <0.05, and ** represents an extremely significant difference at *P* <0.01. The same as below.

Callose acts as a barrier against pathogenic microorganisms. To study the effect of BABA on tobacco callose, the changes in tobacco callose under different treatments were compared (Fig 4). when tobacco was not inoculated with pathogenic fungus, the callose deposition levels were extremely low in water-treated tobacco, but the callose deposition was significantly increased with the BABA treatment. When tobacco is inoculated with *P*. *parasitica*, the callose deposition was significantly higher in the BABA‐treated tobacco plants than the Water‐treated ones. Therefore, we think that BABA-induced resistance to tobacco black shank is related to the accumulation of callose in tobacco cells.

### 3.4 H_2_O_2_ content and *NtrbohD* expression in tobacco

**Fig 5:**
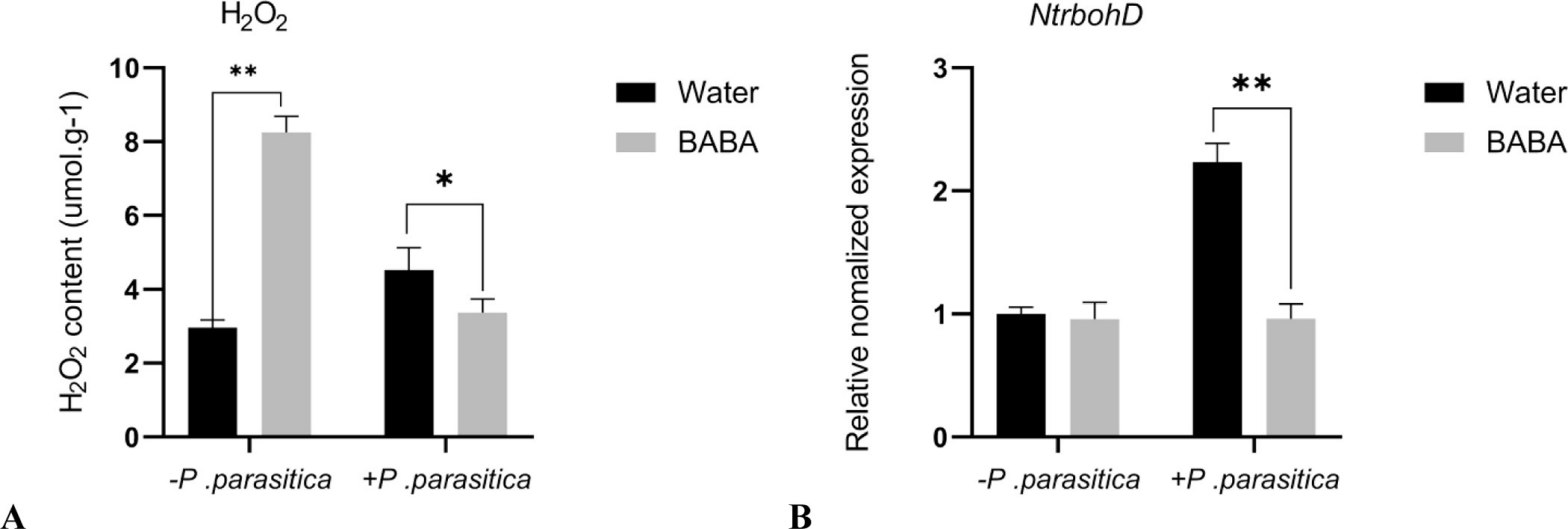
The effect of BABA on the H_2_O_2_ content of tobacco.

To study the potential mechanism of BABA-induced resistance to tobacco black shank, H_2_O_2_ accumulation was compared across different treatments (Fig 5). The H_2_O_2_ content was significantly increased in the BABA-treated tobacco plants compared with Water‐treated plants without the inoculation of *P*. *parasitica*. The results indicated an enhanced ROS burst in the BABA‐treated tobacco plants. The H_2_O_2_ content of BABA-treated tobacco plants decreased significantly by 25.6% compared with Water-treated plants under the inoculation of *P*. *parasitica*. We further quantified the changes in the expression level of *NtrbohD* related to the ROS burst. The qPCR results showed that the transcription level of *NtrbohD* was not significantly changed between the Water‐ and BABA‐treated tobacco plants without *P*. *parasitica* treatment. However, the BABA‐treated tobacco plants had significantly lower expression levels of *NtrbohD* than the water‐treated plants under the inoculation of *P*. *parasitica*, which was consistent with the H_2_O_2_ content results. BABA inhibits the burst of ROS after inoculation with *P*. *parasitica* and reduces the toxic effect of H_2_O_2_ on tobacco plants. These results indicated that BABA may induce resistance to tobacco black shank by enhancing the accumulation of H_2_O_2_ in tobacco plants.

### 3.5 The level of plant hormones and the expression of their related genes

**Fig 6:**
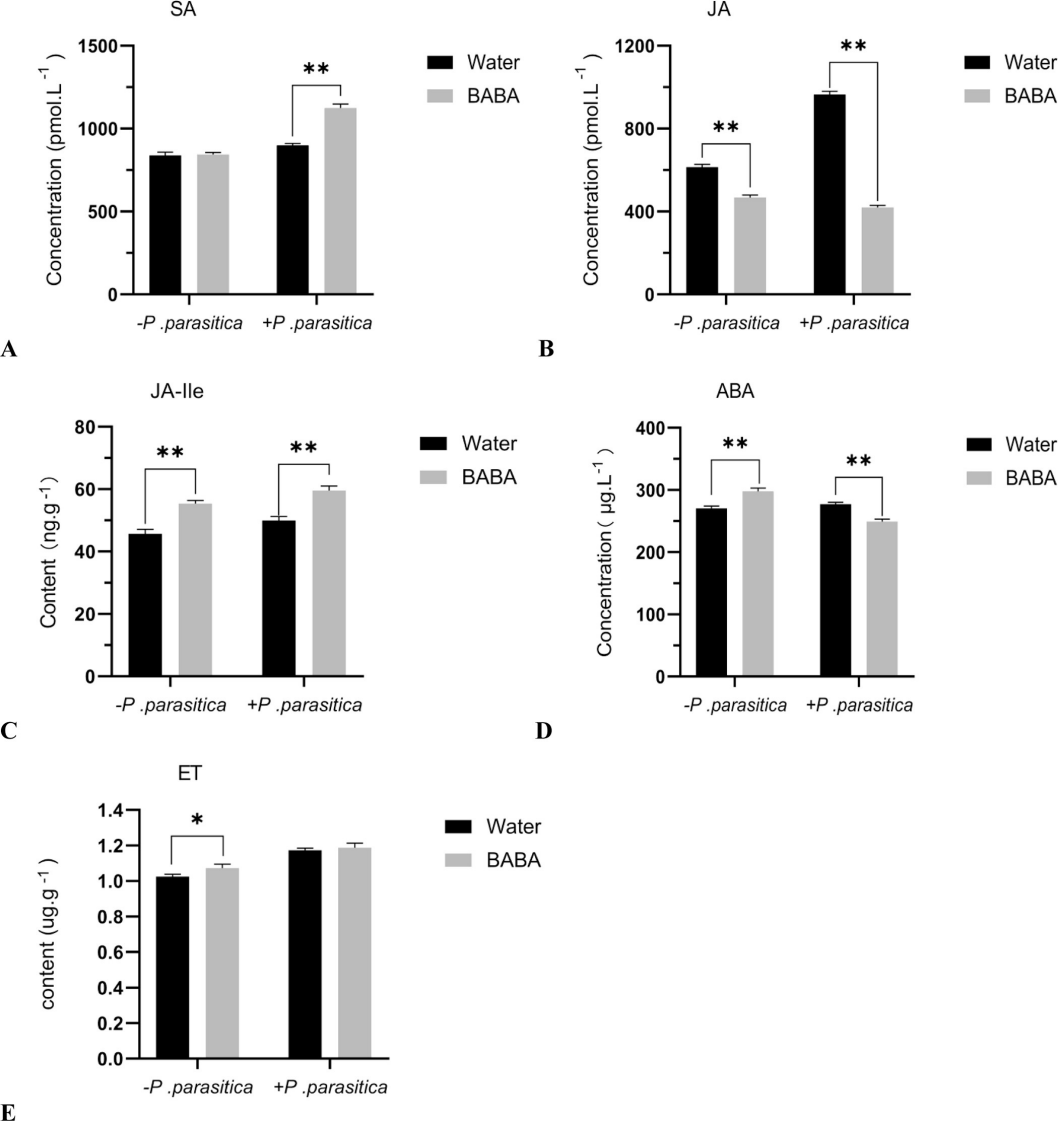
The effect of BABA on the levels of different plant hormones in tobacco.

In order to further study the BABA-induced signal transduction pathway of resistance to tobacco black shank, the levels of plant hormones were quantified in tobacco plants with different treatments (Fig 6). The levels of free SA did not shows significant differences in the Water- and BABA-treated tobacco plants without the inoculation of *P*. *parasitica*. However, compared with the Water-treated ones, free SA levels were significantly higher in the BABA-treated tobacco plants under the inoculation of the *P*. *parasitica*. The BABA‐treated tobacco plants had significantly lower the level of free JA and ABA than that of the Water‐treated ones under the inoculation of the *P*. *parasitica*. The level of JA-Ile was significantly increased in the BABA-treated tobacco plants compared with water‐treated ones with or without the inoculate *P*.*parasitica*. Compared with the Water-treated ones, ET levels were significantly higher in the BABA-treated tobacco plants under the without the *P*. *parasitic*a treatment. However, the levels of ET did not shows significant differences in the Water- and BABA-treated tobacco plants under the inoculation of the *P*. *parasitic*a. The hormone results indicated that BABA may have activated tobacco resistance by enhancing the accumulation of SA and JA-Ile.

**Fig 7:**
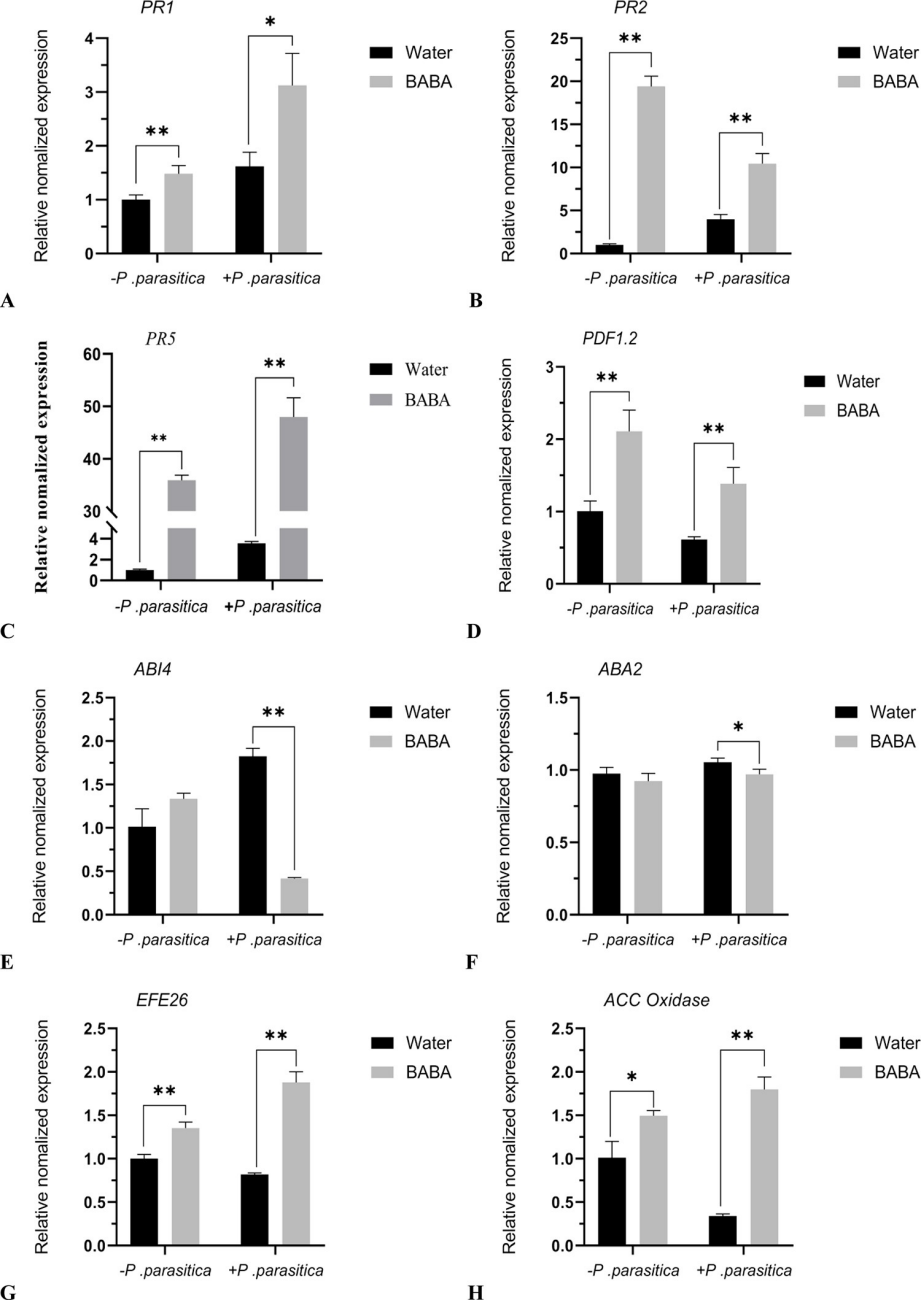
The effect of BABA on the expression of plant hormone related gene in tobacco.

In addition, the transcription levels of several typical genes were quantified in SA, JA, ABA and ET related pathways (Fig 7). the transcription levels of SA‐responsive genes were analyzed, including *PR1*, *PR2*, and *PR5* in higher plants. The expression levels of *PR1*、*PR2*, *PR5* in BABA-treated tobacco plants were significantly higher than Water-ones with or without the inoculate *P*. *parasitica*. The levels of ET did not shows significant differences in the Water- and BABA-treated tobacco plants under the inoculation of the *P*. *parasitica*. However, The expression levels of *ACC Oxidase* and *EFE26* genes related to the ET pathway in BABA-treated tobacco plants were significantly higher than Water-ones. *PDF1*.*2* is a typical gene of JA-response. *ABI4* and *ABA2* are related genes with ABA. The results showed that The expression levels of *PDF1*.*2* was significantly increased in the BABA-treated tobacco plants compared with Water‐treated ones with or without the inoculate *P*. *parasitica*. The expression levels of *ABA2* and *ABI4* in BABA-treated tobacco plants were significantly lower than Water-ones under the inoculation of the *P*. *parasitica*, which is consistent with the level of ABA. Taken together, SA-, JA-, and ET-signaling pathways may contribute to the BABA-induced resistance to tobacco black shank, while ABA may not be very important for the BABA‐induced plant disease resistance.

## 4. Discussion

BABA can induce the resistance of many annual and perennial crops to oomycetes. Oort first discovered that BABA induced the resistance of tomato to late blight infection by *P*. *infestans* in 1960 [19]. Slaughter found that BABA can induce resistance of grapes to downy mildew in 2008 [20]. Subsequently, Walz and Simon found that BABA enhanced cucumber resistance to downy mildew and anthracnose pathogens [21]. Tobacco black shank is an important soil-borne fungal disease. Therefore, in the present study, the related responses and mechanisms of the induction of tobacco resistance to tobacco black shank by BABA were investigated mainly using *P*. *parasitica* as the pathogen.

BABA is an inducer, and the proper concentration is important for effective induction. *P*. *parasitica* was inhibited by 21.78% at 5 mmol^.^L^-1^ BABA. 10 mmol^.^L^-1^ BABA had the strongest inhibition of *P*. *parasitica*, but this level did not reach 50%. In addition, *P*.*parasitica* was not completely inhibited at 20 mmol^.^L^-1^ BABA. This may be due to the ability of *P*.*parasitica* isolates to detoxify BABA. In addition, it was found that when the concentration of BABA was more than 5 mmol^.^L^-1^, BABA had great toxic effects on tobacco leaves. The effect of BABA on tobacco black shank in a greenhouse showed that 5 mmol^.^L^-1^ BABA can effectively alleviate tobacco black shank disease. In view of its efficacy and safety, 5 mmol^.^L^-1^ BABA was considered the optimal concentration to induce resistance to tobacco black shank.

Callose under biotic or abiotic stress plays a regulatory role in plant life activities. Plant cell wall and related structures, such as plasmodesmata, regulate the bottleneck of plasmodesmata through the deposition and degradation of calcium loss, contraction or expansion, so as to cope with biological stress caused by mechanical damage, various pathogens and insects [22, 23]. It is generally believed that BABA induces callose formation, papilla formation or cell lignification in plant cell walls, which can prevent infection and enhance disease resistance [24–26]. In this study, a large amount of callose deposition was observed in BABA-induced tobacco. Therefore, We think that callose deposition also plays an important role in BABA-induced tobacco resistance to tobacco black shank.

When plants are under abiotic and biotic stresses during their growth, the burst of reactive oxygen species (ROS) is one of their fastest defense responses. ROS play an important role in the immune process of plants [27, 28]. NADPH oxidases, members of the respiratory burst oxidase homolog (RBOH) family, are very important in ROS production. NADPH oxidase can transfer electrons from NADPH to O_2_ to form O_2_^−^, which further reacts to form H_2_O_2_ [27–29]. *NtrbohD* is a gene encoding NADPH oxidase in tobacco; it has been proven to be able to produce ROS when tobacco cells are affected by the fungal inducer Cryptogein [30]. In this study, the accumulation of H_2_O_2_ may contribute to BABA-induced plant disease resistance. However, the BABA‐treated tobacco plants had significantly lower H_2_O_2_ content and expression levels of *NtrbohD* than the Water‐treated plants under the inoculation of *P*. *parasitica*. This is contrary to the result of Peng Qin [31]. The reason for this discrepancy may be that the test sampling time is different, and production of reactive oxygen species under pathogen infection is reported to create oxidative stress [32]. The BABA-induced antioxidant enzyme system balances the content of H_2_O_2_ and the expression of *NtrbohD* and alleviates the oxidative damage of reactive oxygen species in plants.

After the plants were treated with an inducer, the disease-resistant signals were amplified through complex signal transduction pathways, which stimulated the plants to produce defensive responses and finally obtain resistance to pathogens. Plant disease resistance signaling pathways mainly include the SA pathway, JA pathway and ET pathway [33, 34]. Generally, SA mainly regulates resistance to hemibiotrophic and biotrophic pathogens. JA and ET play a more important role in plant defense against necrotrophic pathogens [33]. ABA regulates many complicated physiological processes, such as seed germination, root growth inhibition and stomatal closure. In addition, ABA also plays a role in biological stress [35, 36]. In this study, SA-, JA-, and ET-signaling pathways may contribute to BABA-induced resistance to tobacco black shank. Some scholars found that BABA-induced resistance to Oomycetes was not dependent on the SA signaling pathway but that induced resistance to TMV, bacteria and *Botrytis cinerea* was mediated by SA signaling [8, 37]. Our results are inconsistent with this finding. The reason for this discrepancy may be that different plants and pathogens invoke different resistance mechanisms to BABA induction. However, there was no accumulation of JA in the process of BABA-induced resistance to tobacco black shank. The possible reason for this phenomenon is that the sampling time of this experiment was too long, and JA forms JA-Ile with isoleucine under the catalysis of JAR1 when plants are subjected to external stimulation. ABA is usually a negative regulator in plants that helps them resist the infection of necrotrophic and biotrophic pathogens [33]. ABA pathways may not be involved in BABA resistance to tobacco black shank.

We found that BABA activated resistance to tobacco black shank by increasing H_2_O_2_ accumulation, callose deposition, SA and JA-Ile production and SA-, JA-, and ET-signaling pathways in the plants. This study helps us understand the possible mechanism by which BABA induces resistance to tobacco black shank and provides a theoretical basis for the application of BABA in tobacco.

## Supporting information

S1 Table The effect of different treatments on gene expression in tobacco. (XLSX)
